# Aesthetic Surgical Approach for Bone Dehiscence Treatment by Means of Single Implant and Interdental Tissue Regeneration: A Case Report with Five Years of Follow-Up

**DOI:** 10.1155/2016/1236310

**Published:** 2016-03-16

**Authors:** Giorgio Lombardo, Jacopo Pighi, Giovanni Corrocher, Anna Mascellaro, Jeffrey Lehrberg, Mauro Marincola, Pier Francesco Nocini

**Affiliations:** ^1^Clinic of Dentistry and Maxillofacial Surgery, University of Verona, Piazzale Ludovico Antonio Scuro 10, 37100 Verona, Italy; ^2^Department of Biomaterials, Implant Dentistry Centre, 501 Arborway, Jamaica Plain, Boston, MA 02130, USA; ^3^Universidad de Cartagena, Avenida del Consulado, Calle No. 30, No. 48-152, Cartagena, Bolívar, Colombia

## Abstract

The replacement of single anterior teeth by means of endosseous implants implies the achievement of success in restoring both aesthetic and function. However, the presence of wide endoperiodontal lesions can lead to horizontal hard and soft tissues defects after tooth extraction, making it impossible to correctly place an implant in the compromised alveolar socket. Vertical augmentation procedures have been proposed to solve these clinical situations, but the amount of new regenerated bone is still not predictable. Furthermore, bone augmentation can be complicated by the presence of adjacent teeth, especially if they bring with them periodontal defects. Therefore, it is used to restore periodontal health of adjacent teeth before making any augmentation procedures and to wait a certain healing period before placing an implant in vertically augmented sites, otherwise risking to obtain a nonsatisfactory aesthetic result. All of these procedures, however, lead to an expansion of treatment time which should affect patient compliance. For this reason, this case report suggests a surgical technique to perform vertical bone augmentation at a single gap left by a central upper incisor while placing an implant and simultaneously to regenerate the periodontal attachment of an adjacent lateral incisor, without compromising the aesthetic result.

## 1. Introduction

Implant therapy was first introduced for the rehabilitation of completely edentulous jaws and has become today a viable option for the rehabilitation of partial and single edentulism [1]. In the anterior maxilla, a successful implant procedure requires not only well-anchored implants, but also natural looking result. The only way to gain this result is to correctly place implants in all the three dimensions of bone (i.e., apicocoronal, faciolingual, and mesiodistal) [2]. Unfortunately, this cannot always be achieved with dental implants due to the presence of alveolar defects. Several clinical and histologic studies have shown the dynamic resorptive process that unfolds after tooth extraction [3]. When the alveolar site presents deficiencies, many techniques can be used for its development. Vertical ridge augmentation has shown to be effective with several techniques, such as onlay autografts or particulate autogenous and xenogeneic bone covered by nonresorbable membranes [4–6]. Paramount to the success of vertical ridge augmentation are five crucial factors: biocompatibility, cell occlusion, space-provision, tissue integration, and ease of use [7].

Most of the published studies report on the outcomes of augmentation procedures in completely edentulous areas. The surgical management of edentulous ridges involves a simpler management of soft tissues in order to obtain a primary wound closure and reduce the risk of bacterial contamination. Alternatively, the presence of teeth in close proximity to the atrophic alveolar ridge can affect the procedure, in particular when teeth present periodontal morbidity [8]. The presence of horizontal defects on implant adjacent teeth limits the possibility of interproximal soft tissue overgrowth between teeth and implants, leading to the need to regenerate the lost periodontal attachment. While ridge augmentation has proven to be effective and predictable, supra-alveolar periodontal regeneration has not yet been demonstrated. Therefore, in clinical practice, periodontal regeneration should precede alveolar augmentation and implant placement. Furthermore, although vertical bone regeneration should occur, it is impossible to predict the amount of resorption of the grafted material, thus leading to the risk of finding part of the implant surface exposed after a certain period of time, if bone augmentation and implant placement are performed simultaneously.

Few reports can be found concerning the feasibility of regenerating a lost periodontal attachment around a tooth, while at the same time performing a vertical ridge augmentation to allow for the delayed placement of an implant [9].

The case report presented here illustrates a technique to regenerate the lost periodontal attachment, improve bone volumes, and allow the simultaneous placement of an implant in the adjacent edentulous alveolar ridge in order to achieve a good aesthetic result in a short rehabilitation time.

## 2. Case Presentation

**Figure 1:**
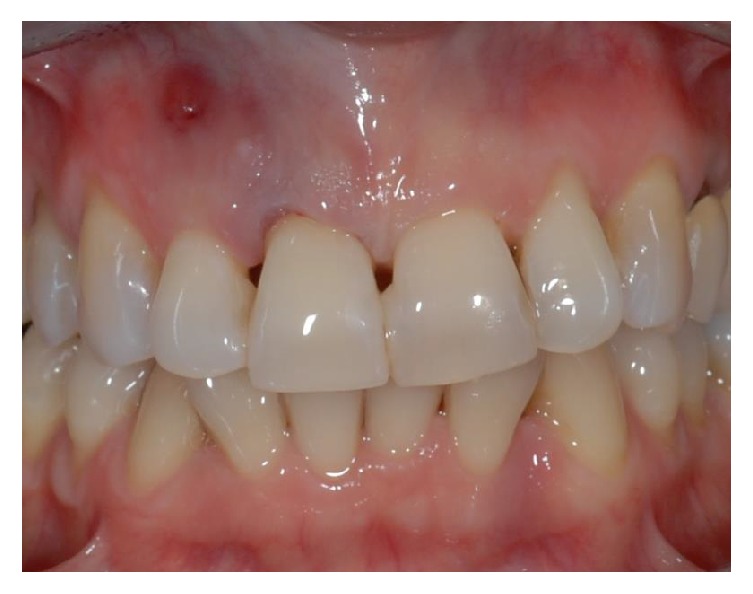
Front side view of patient upon presentation.

**Figure 2:**
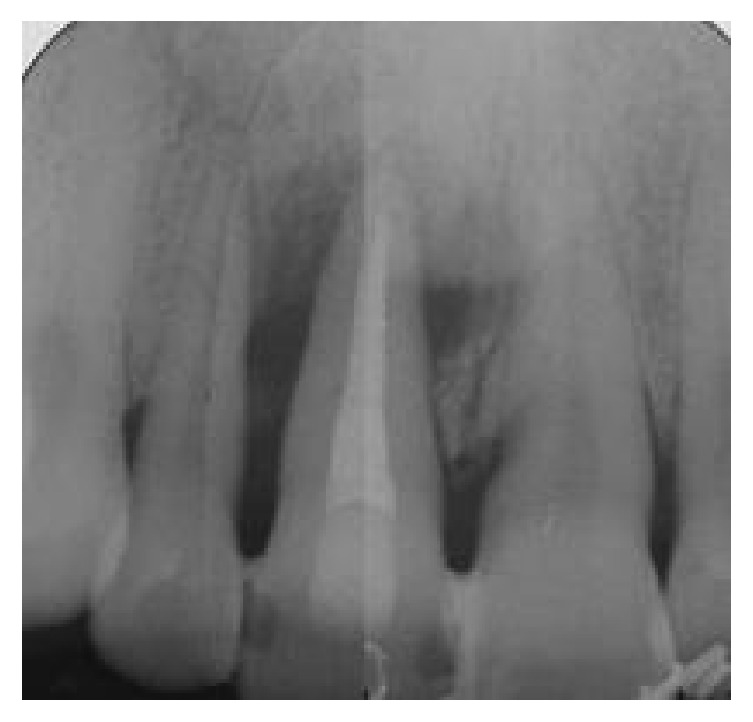
Intraoral periapical radiograph showing bone loss around the right central incisor and involving the mesial alveolar wall of the right lateral incisor.

**Figure 3:**
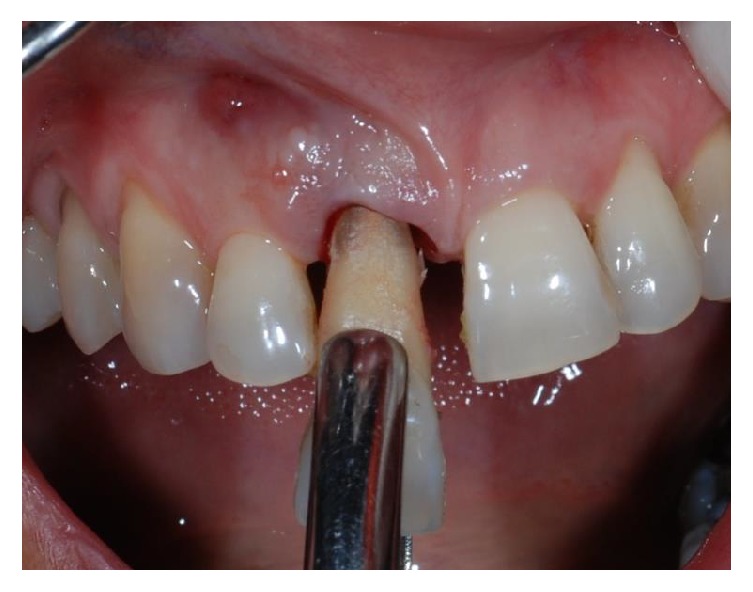
Plaque and calculus can be observed on the root surface at the time of extraction, performed with minimal trauma.

**Figure 4:**
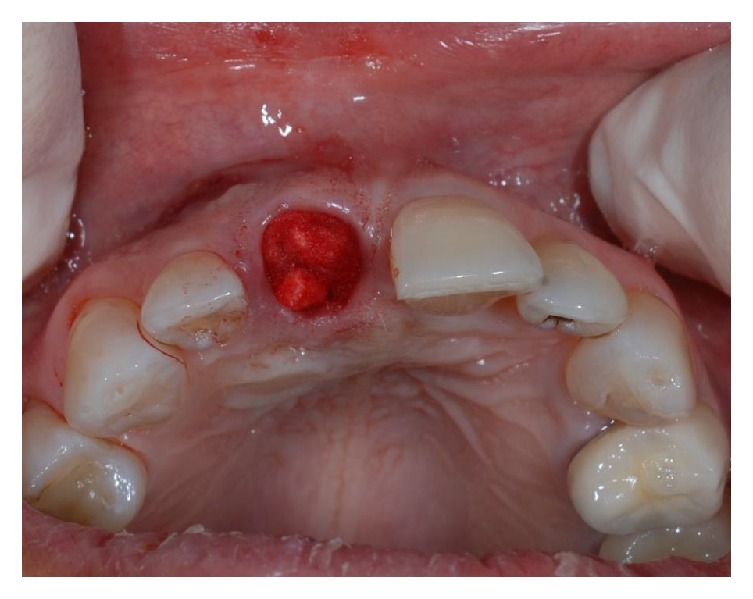
Occlusal view of the fresh alveolar socket after extraction and application of Spongostan*™*.

**Figure 5:**
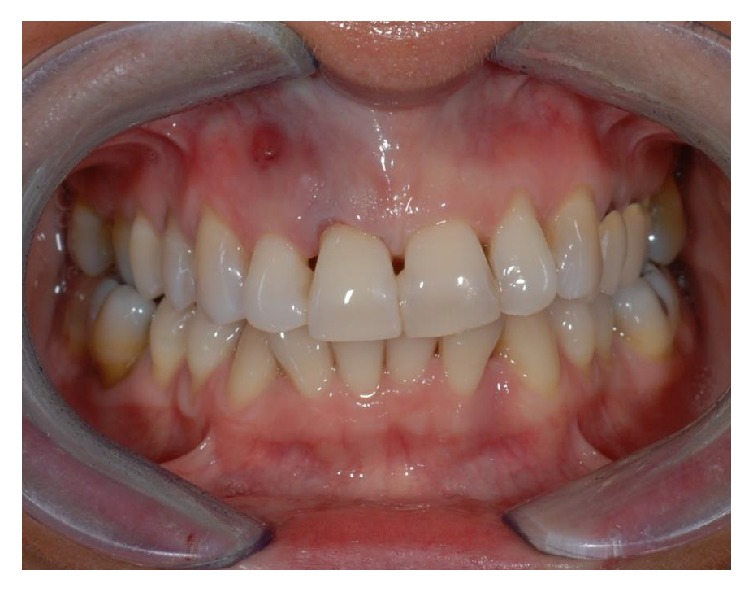
The crown of the extracted tooth was splinted to the adjacent elements and used as provisional pontic.

**Figure 6:**
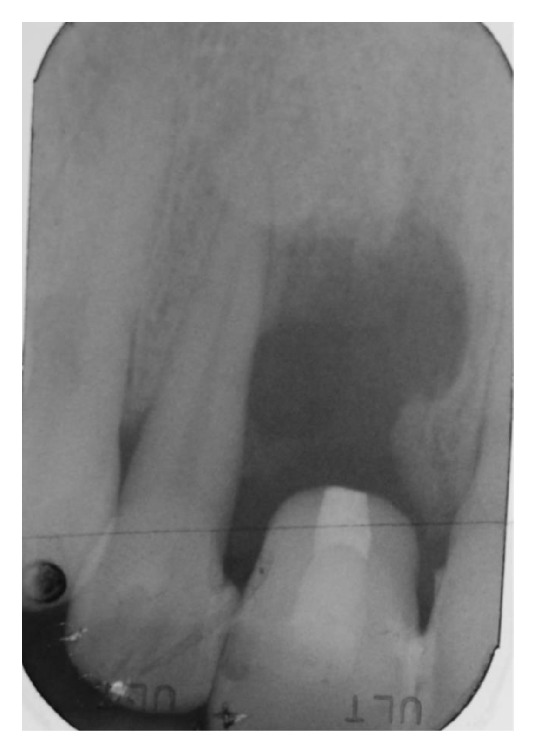
Postoperative X-ray after tooth extraction.

A 43-year-old woman was referred to us presenting a hopeless upper left central incisor, affected by a large endoperiodontal lesion involving the buccal wall of the alveolar crest and the mesial aspect of the neighboring lateral incisor (Figures 1 and 2). The patient accepted the proposal of extracting the tooth and to replace it with an implant. The extraction was performed with minimal trauma and the extraction socket was filled with absorbable hemostatic gelatin sponge (Spongostan*™* Dental, Ethicon, Edinburgh, UK) (Figures 3 and 4). The root of the extracted tooth was cut out and the remaining crown was used as provisional pontic element until the implant would have been placed (Figures 5 and 6).

**Figure 7:**
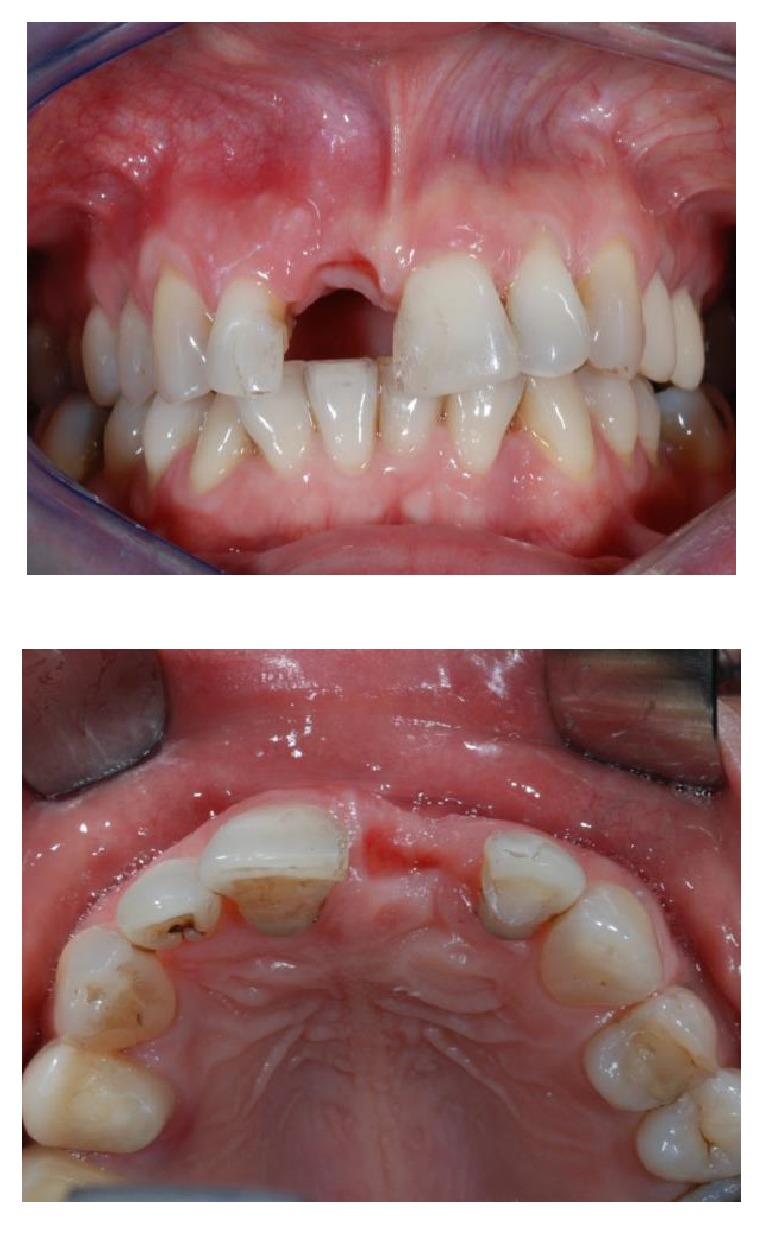
Frontal (a) and occlusal (b) views after six weeks of healing.

**Figure 8:**
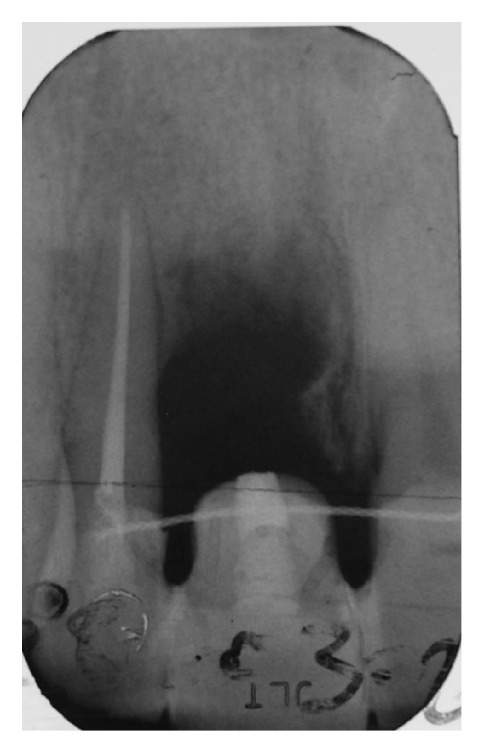
The radiograph taken before implant insertion shows a complete loss of the buccal and palatal bone.

**Figure 9:**
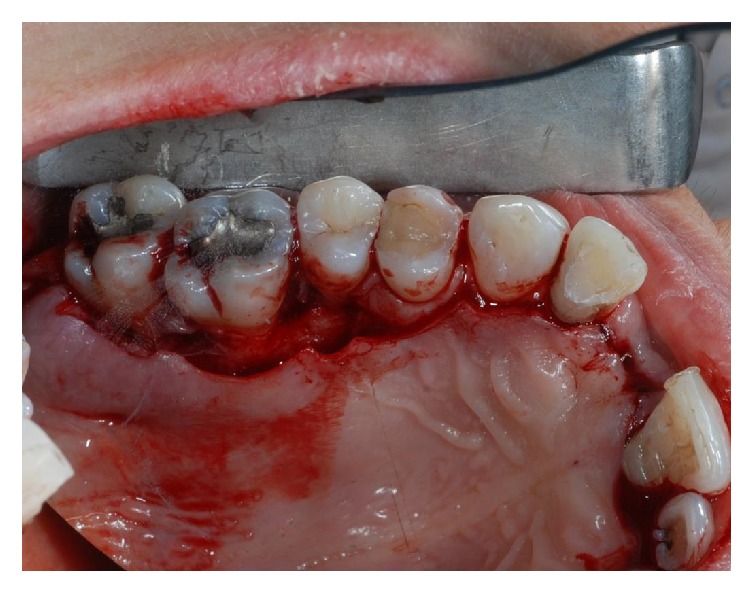
Occlusal view of the surgical incision at implant insertion time. A full thickness flap at the single gap continued with a partial thickness flap to harvest a connective tissue graft.

**Figure 10:**
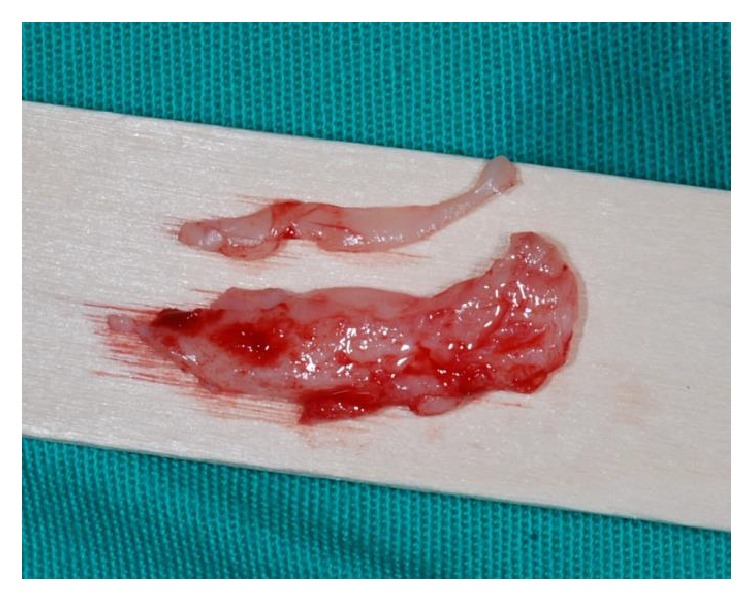
Aspect of the harvested connective tissue graft.

**Figure 11:**
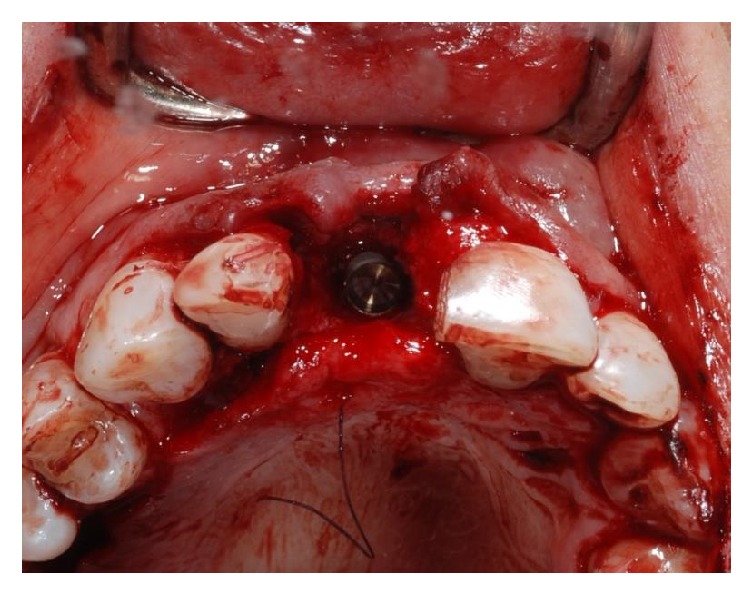
Surgical placement of the Bicon implant. The implant lies 1 mm below the margin of the buccal bone dehiscence.

**Figure 12:**
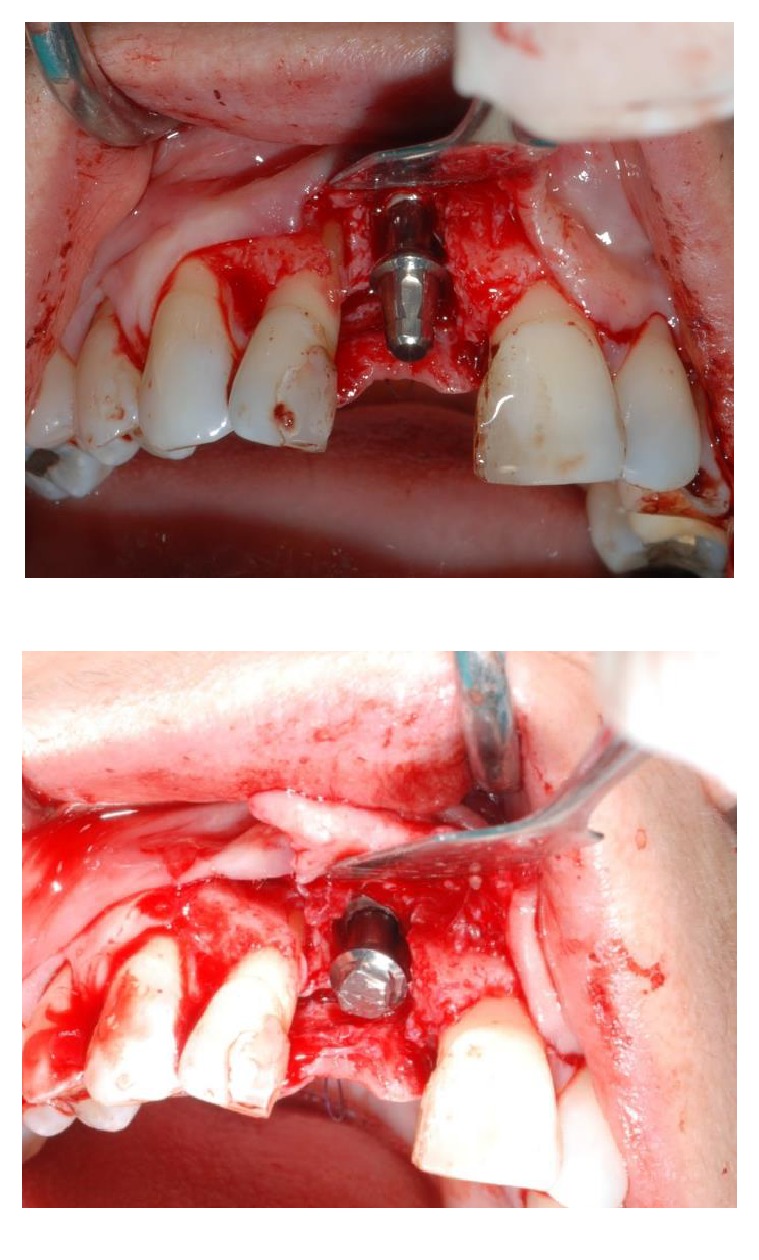
A stealth abutment was tested (a) and then modified on its top (b) to primarily close the wound maintaining a proper bone and mucosal tunnel.

After six weeks of healing soft tissues were well conditioned by the provisional pontic (Figures 7(a) and 7(b)) but a large bone defect was radiographically noticed (Figure 8). A full thickness flap was raised at the edentulous ridge and was continued on the palatal side with a partial thickness flap till the last molar (Figure 9), from where a free connective tissue graft was harvested (Figure 10). The alveolar defect was degranulated and the root surfaces of the adjacent elements were decontaminated using ultrasonic tips and then polished with low abrasive burs (Intensiv Perio Set®, Intensiv SA, Montagnola, Switzerland). After that a 5.0 × 8.0 mm, plateau design, locking-taper implant (Bicon LLC, Boston, MA) was placed under the buccal margin of the bone dehiscence (Figure 11). To maintain a proper bone and mucosal tunnel, a stealth abutment was modified on its top and then connected to the implant (Figures 12(a) and 12(b)), and in the meantime the connective graft was fixed to the buccal flap.

**Figure 13:**
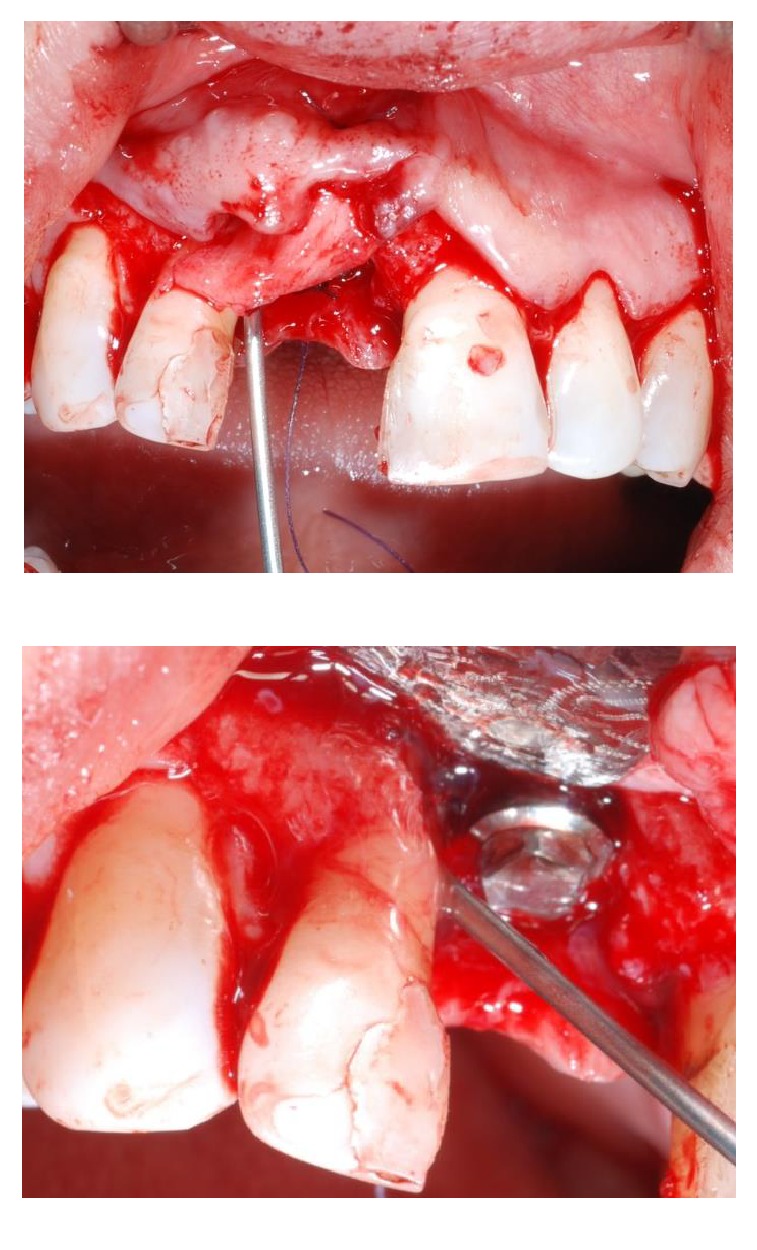
After fixation of the connective tissue graft to the buccal flap (a), Emdogain® was applied on the lateral incisor root surface to regenerate the lost periodontal attachment (b).

**Figure 14:**
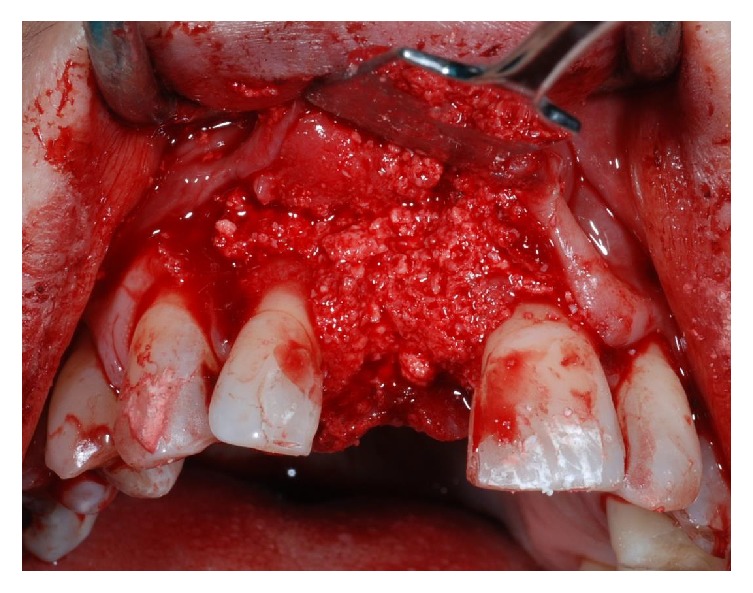
Bio-Oss® was applied to cover the defect around the implant.

**Figure 15:**
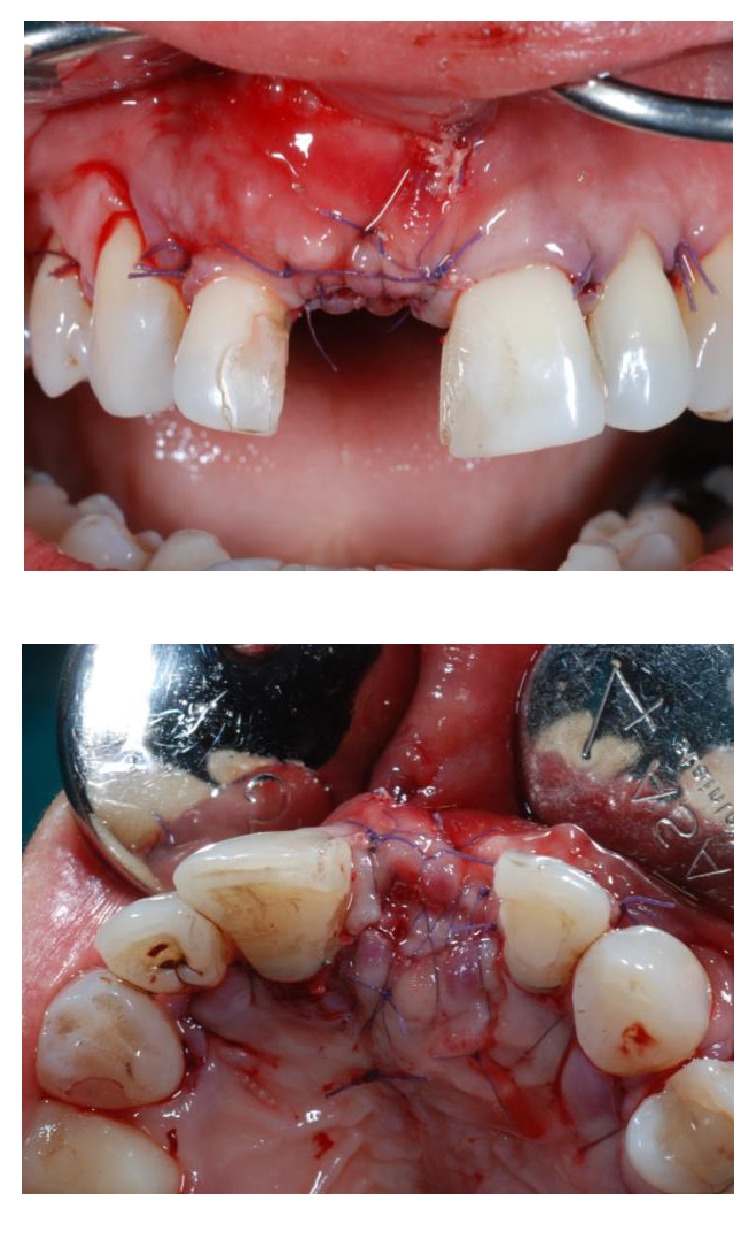
Frontal (a) and occlusal (b) view at the end of the surgical intervention.

**Figure 16:**
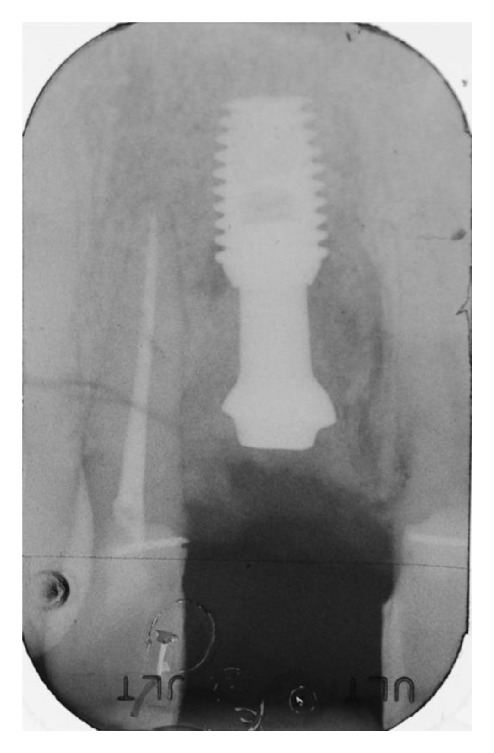
Postoperative X-ray shows fulfilling of the defect, with the implant lying in native bone below the grafted material.

**Figure 17:**
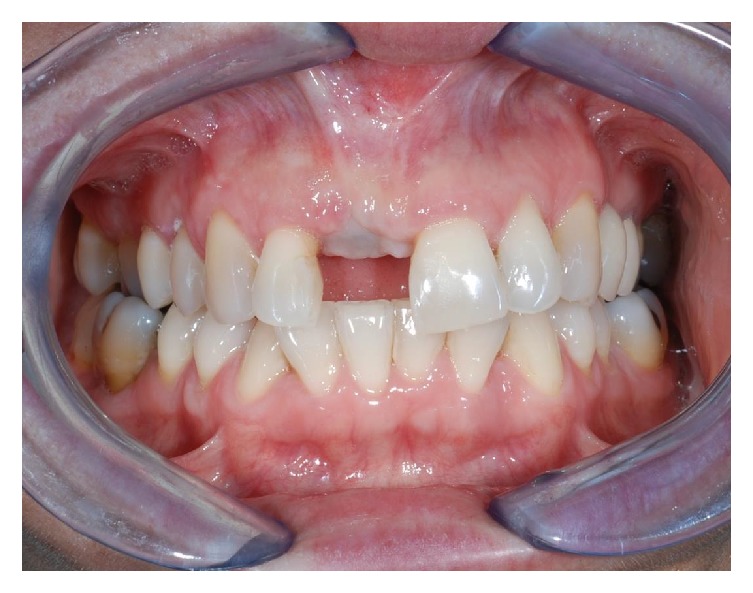
Frontal view following provisional pontic removal, after four months of healing.

**Figure 18:**
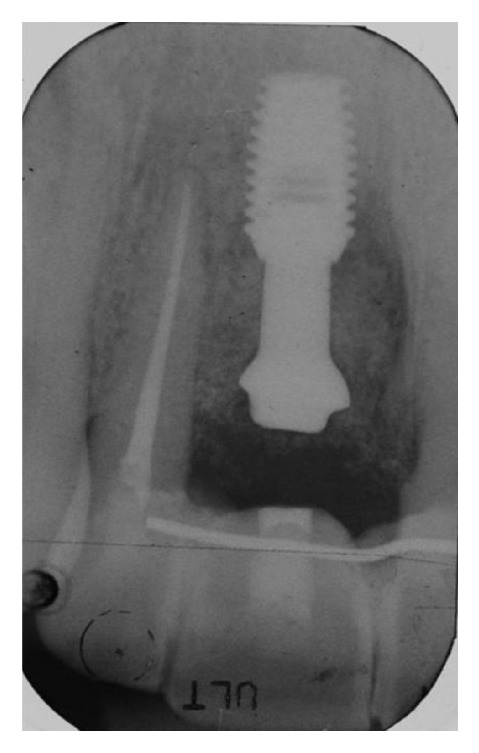
Radiograph at four months of healing showing good integration of the implant and the grafted material.

**Figure 19:**
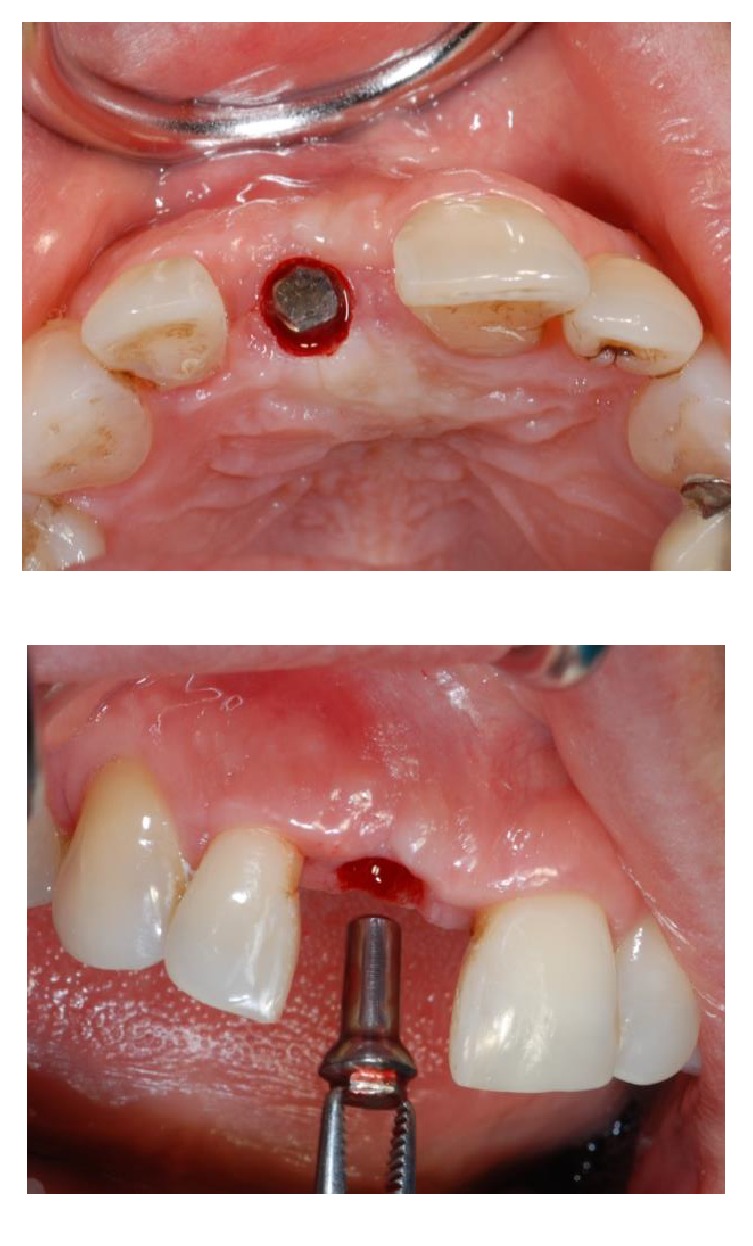
After performing a circular incision (a), the provisional stealth abutment was removed (b).

**Figure 20:**
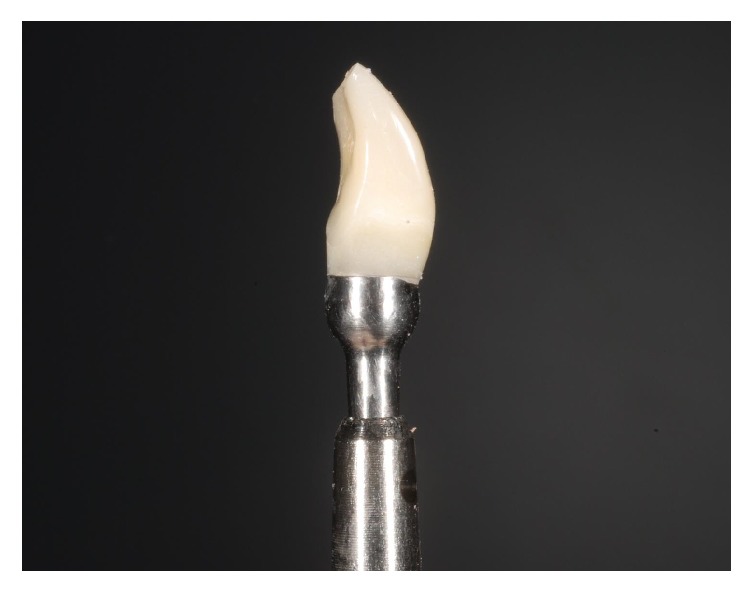
The provisional integrated abutment crown before fixation.

**Figure 21:**
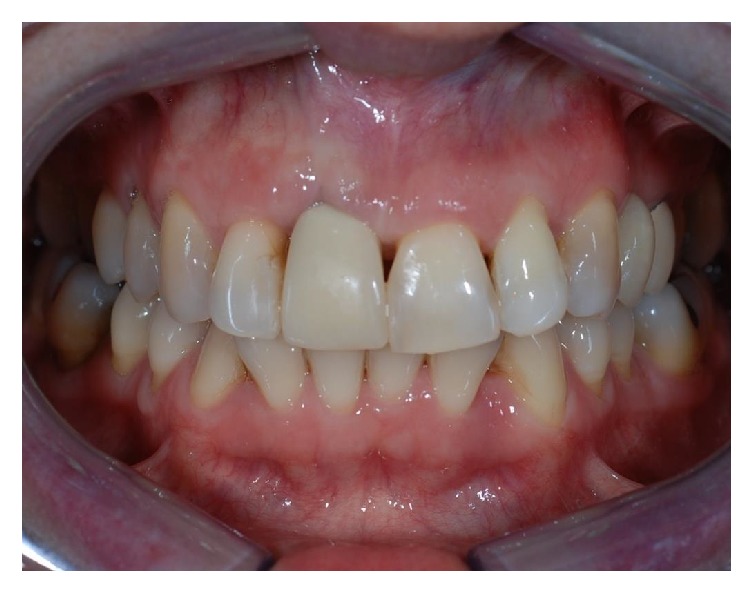
Frontal view after application of the provisional integrated abutment crown.

**Figure 22:**
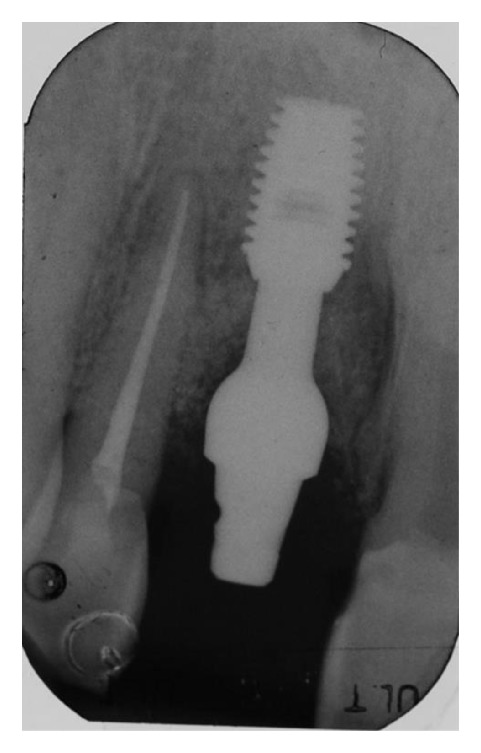
Radiograph showing the long abutment post, chosen to respect the bone and mucosal tunnel.

**Figure 23:**
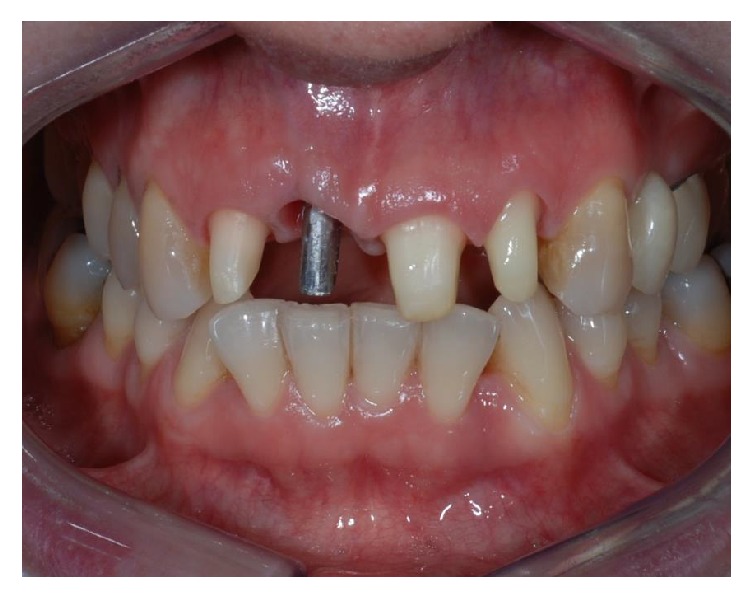
Frontal view before prostheses delivery. The implant abutment was tested before extraoral cementation of the crown.

**Figure 24:**
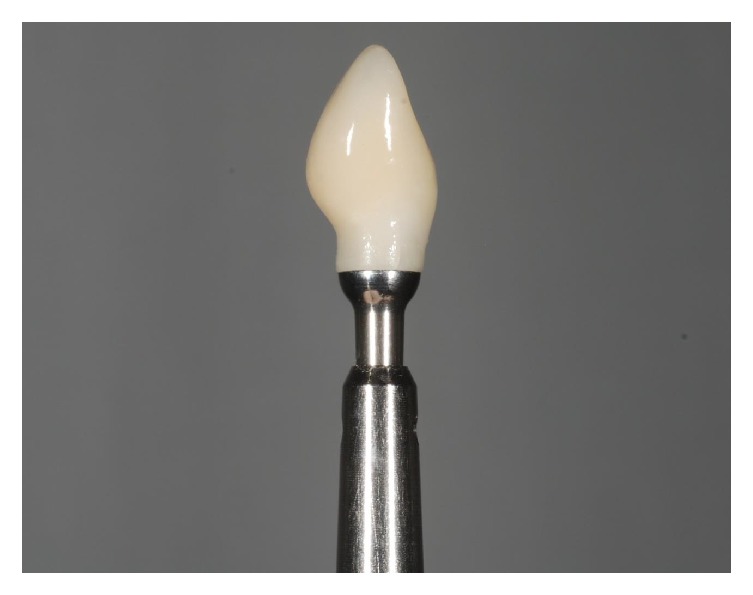
Aspect of the implant crown after extraoral cementation.

**Figure 25:**
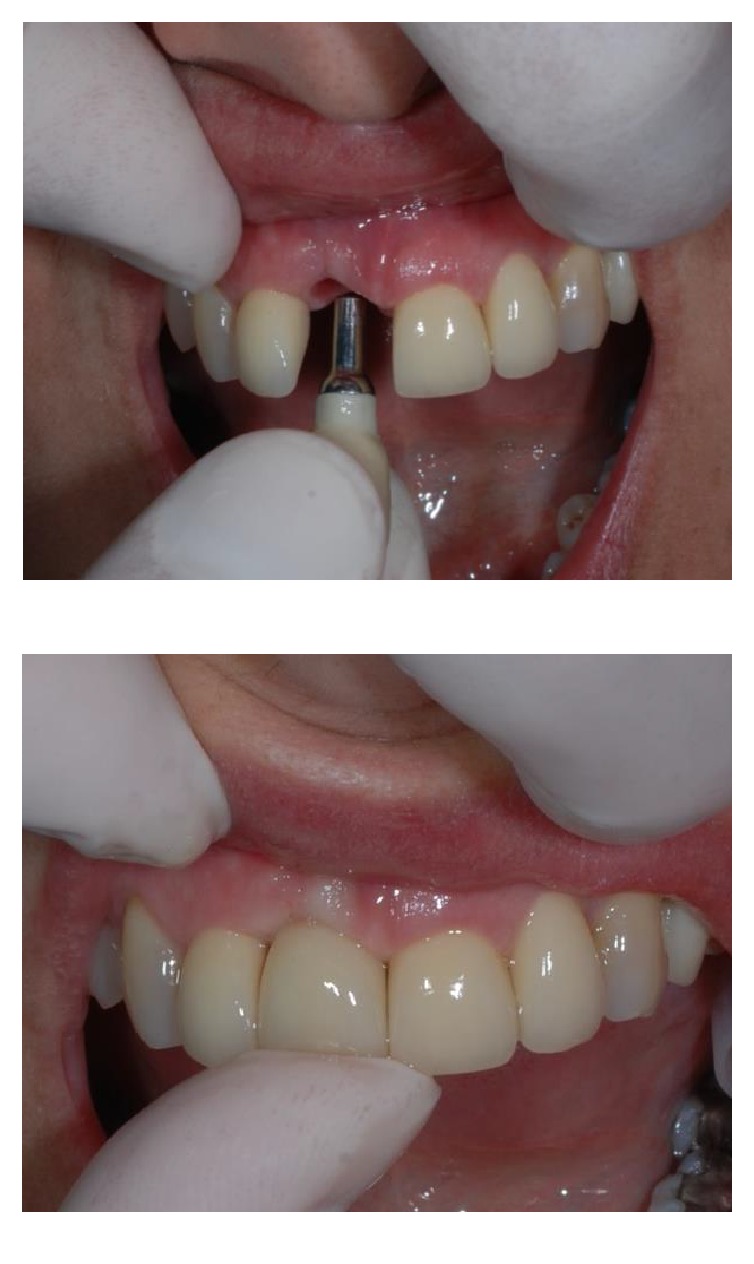
The abutment and crown were inserted through the long axis of the implant well (a) and then manually pressed (b) before tapping with mallet.

**Figure 26:**
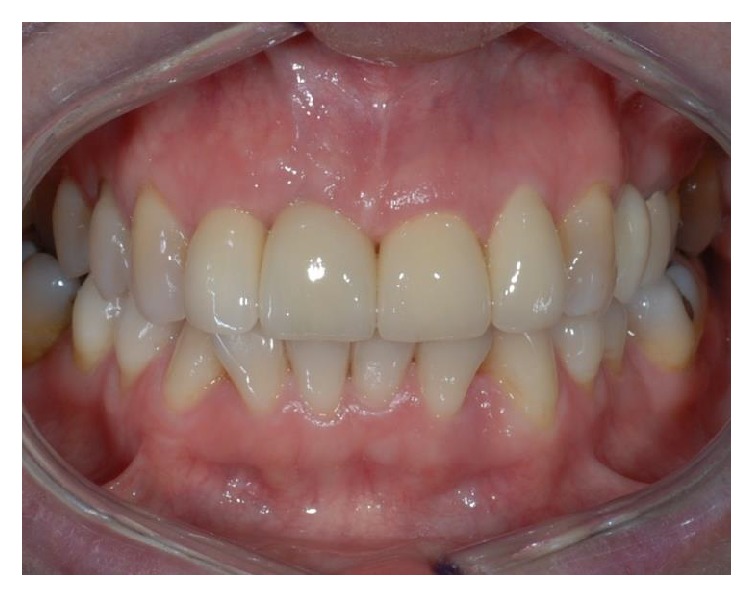
Frontal view after prostheses delivery.

**Figure 27 fig27:**
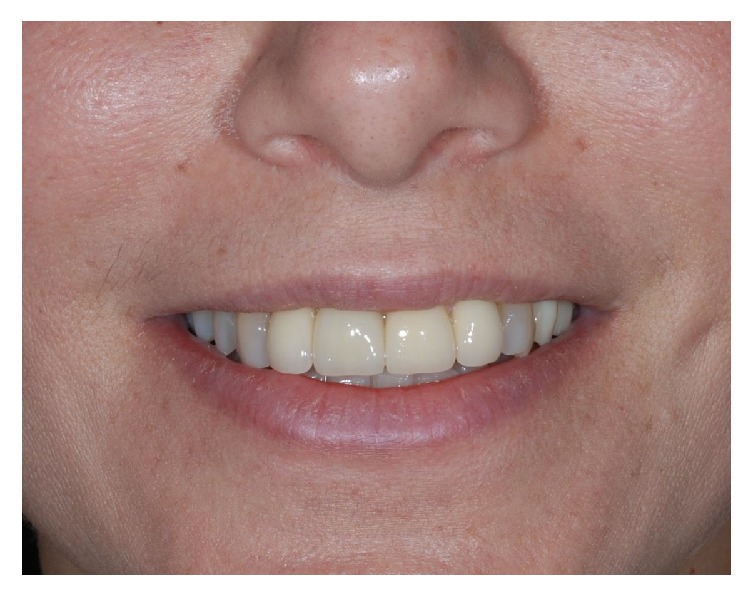
The patient's natural looking smile at the end of the therapy.

**Figure 28:**
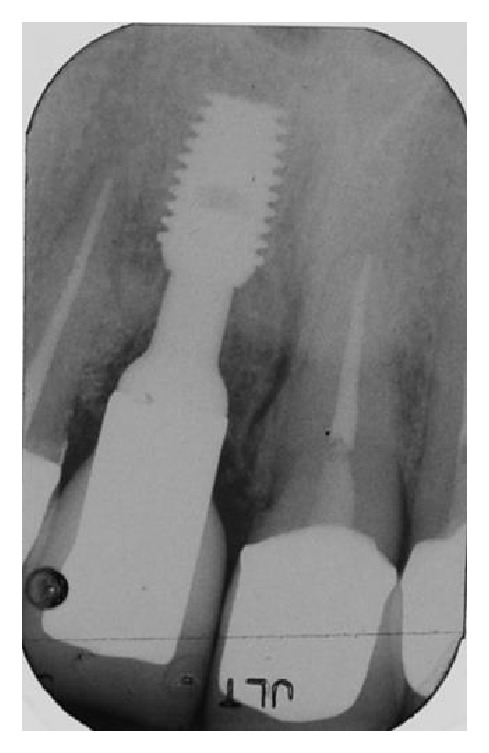
Control X-ray at the end of therapy.

Following implant placement, the connective tissue graft was fixed to the buccal flap, and the compromised root surface of the lateral incisor was treated with a regenerative approach, using enamel matrix derivatives (Emdogain®, Straumann, Basel, Switzerland) (Figures 13(a) and 13(b)). A bovine xenograft material (Bio-Oss Collagen®, Geistlich Pharma, AG, Wolhusen, Switzerland) was grafted around facial and buccal aspects till the top of the implants abutment (Figure 14). The connective tissue graft was used as a membrane, over which the buccal flap was advanced and sutured to obtain a primary wound closure without tension (Figures 15(a) and 15(b)). After taking a postoperative radiograph (Figure 16), the provisional pontic was placed again and the patient was suggested to take oral antibiotics (Augmentin®, GlaxoSmithKline, UK) and to rinse the mouth twice a day using 0.2% chlorhexidine mouthrinse. Postoperative controls, during which professional oral hygiene was always performed, were made at one and two weeks and then once a month for four months. After four months, the implant and the grafting material appeared well integrated (Figures 17 and 18), so a small circular incision was made to allow for the removal of the customized abutment (Figures 19(a) and 19(b)), and a provisional integrated abutment crown was connected (Figures 20 and 21). To respect the new regenerated hard interproximal tissues, we chose an abutment with a long post (Figure 22). Six months after the contralateral incisors were endodontically treated and then reduced to abutment along with the right lateral incisor (Figure 23). Lateral/anterior protrusion of the definitive restorations was achieved using canine guidance: in this way, mutually protected occlusion prevented contact between incisors during all mandibular eccentric movements, and incisors came into contact with their antagonists only during maximum intercuspation. To orient the seating of the final abutment, a jig was fabricated and utilized to aid in correct positioning. A direct impression was then taken using a polyether impression material (Impregum Penta, 3MESPE, St. Paul, MN). A stone cast using type IV extra-hard dental stone was then prepared, from which the definitive abutment could be individually modified. Finally, four zirconia crowns were fabricated. The implant crown was cemented on the abutment using extraoral cement (RelyX Unicem, 3M ESPE, St. Paul, MN) (Figure 24). The abutment and crown were then tapped through the long axis of the post into the implant well using a 250 g mallet (Figures 25(a) and 25(b)). The other crowns were cemented on the natural abutments using a zinc phosphate definitive cement (Harvard Cement®, Harvard Dental International, Hoppegarten, Germany) (Figures 26 [Fig fig27]–28).

## 3. Results

**Figure 29:**
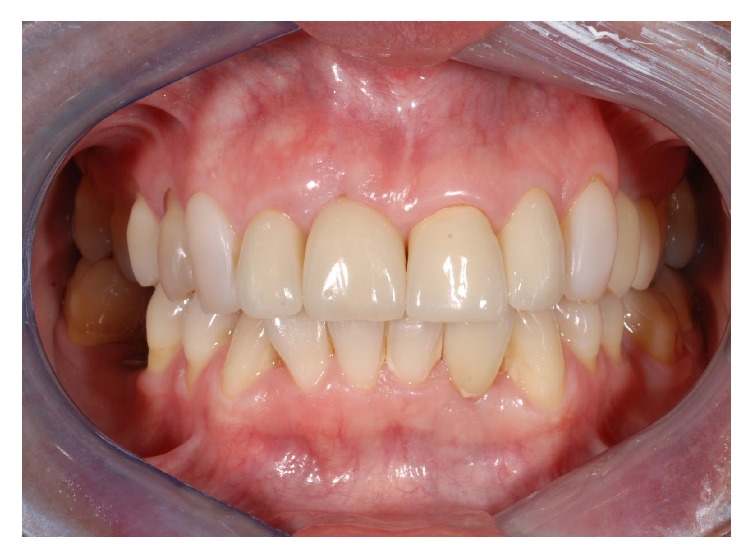
Frontal view showing the aesthetic result at the five-year follow-up examination.

**Figure 30:**
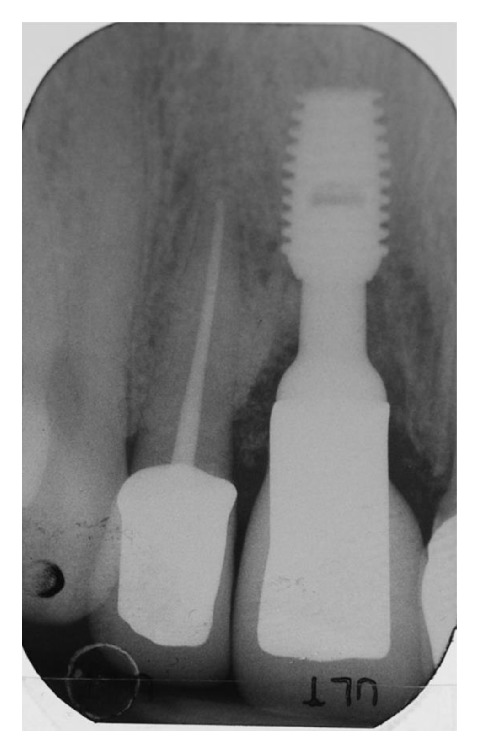
The radiograph performed after five years shows full integration of the implant and good stability of interproximal soft tissues, along with almost full filling of the lateral incisor's periodontal defect.

The patient returned to our observation after five years of prosthetic loading. The clinical follow-up examination revealed a satisfying aesthetic result, with adequate contours of peri-implant soft tissues and an almost complete filling of interproximal spaces (Figure 29). The periapical radiograph obtained showed the implant completely integrated in native bone, while new bone was visible around the implant abutment until the margins of the ceramic crowns (Figure 30).

The peri-implant tissues surrounding the implant were found to be in healthy conditions, free from any sign of inflammation and not bleeding after probing. Furthermore, the right lateral incisor presented probing depths below 5 mm, without bleeding after probing.

The patient concluded that she was fully satisfied with both the aesthetic and functional results of the procedure.

## 4. Discussion

In the present case report, the regeneration of a horizontal periodontal defect was achieved while simultaneously placing an implant and performing a vertical ridge augmentation. Success of periodontal regeneration usually depends on the initial probing depth in relation to the amount of the intrabony/vertical component [10]. In general, effectiveness of enamel matrix derivatives in inducing periodontal regeneration of intrabony defects was proven to be almost equal if compared to guided tissue regeneration [11]. On the other hand, outcomes of regenerative therapy of periodontal supra-alveolar defects are known to be unpredictable, and there is little evidence to support such a procedure [4–6, 12]. In a recent animal trial, enamel matrix derivatives were combined with a scaffold material to obtain successful supracrestal bone regeneration around implants [13]. Also, with respect to vertical ridge augmentation, it is not possible to predict the amount of vertical bone gain; therefore, for both the procedures, we sought the use of a barrier able to provide stable space for tissue regeneration against the pressure made by the uppermost soft tissues and to avoid the migration of epithelial cells. Under this point of view, however, both nonresorbable and resorbable barriers have resulted in the same performance [14]. Despite this, a disadvantage of using nonresorbable barriers is that they need a second intervention for their removal. Membranes, however—particularly nonresorbable membranes—are susceptible to exposure during the healing-phase, leading to only a partial regeneration. Assuming that the placement of an implant occurred followed by membrane exposure, then the aesthetic appearance of soft tissues might be negatively affected, thus compromising the overall success of implant therapy in the anterior maxilla.

Tooth loss in aesthetic areas has a profoundly negative impact on the patient's social life, and attempts to reduce rehabilitation times were of primary concern; for this reason, a simultaneous approach for periodontal regeneration, alveolar regeneration, and implant insertion was attempted.

Therefore, to reduce rehabilitation time, we forgo the use of membrane and we used a subepithelial free connective tissue graft to protect the underlying grafting material and to prop up peri-implant soft tissues. The use of free or pedicle soft tissue grafts to preserve gingival color and tissue characteristics is supported by the literature [15–17]. Moreover, connective tissue grafts have proven their capability of performing an effective barrier function [18–20].

Vertical ridge augmentation was also performed due to the presence of the customized abutment connected to the implant at the time of its insertion, which maintained the space for grafting material preventing any possible collapse due to compression. As a result of the subcrestal placement and vertical ridge augmentation, the abutment post had to cross a long transmucosal tunnel to connect to the implant. Implant subcrestal placement should lead to further bone resorption in presence of microgaps located at the implant-abutment connection. However, the literature has shown that some locking-taper implant-abutment connections, like the one used in this study, are resistant to bacterial leakage [21, 22], and some studies have demonstrated minimal bone resorption around similar implants placed subcrestally [23, 24].

While the sloping shoulder design of the implant used provides space for interproximal bone regeneration, only histological analysis could distinguish the presence of new bone or simply a filled defect. Nevertheless, the absence of inflammation, along with radiological findings, indicates that the material is well integrated and does not induce a deleterious immunological response. Furthermore, the implant was placed in native bone immediately under the buccal ridge of the alveolar defect, in order to prevent complications if graft resorption occurred. This means that the regenerated hard tissue served to give support to peri-implant soft tissues, to ensure their stability, and to improve the periodontal attachment of the lateral incisor. Finally, for the case shown here, a time period of five years has shown to be sufficient to permit the stability of peri-implant tissues using this aforementioned approach.

To the best of our knowledge, this is the first report concerning the simultaneous application of periodontal and alveolar regeneration at the implant surgery stage. Based on the positive aesthetic and functional results obtained with the subcrestal placement of a locking-taper connection implant in the anterior maxilla, we look forward to further investigate the efficacy of this treatment as a therapeutic approach.
